# Salt as a Trigger for Atrial Tachycardia/Fibrillation

**DOI:** 10.7759/cureus.26168

**Published:** 2022-06-21

**Authors:** Jerome Goddard, Cori J Speights, Mark Borganelli

**Affiliations:** 1 Department of Biochemistry, Molecular Biology, Entomology, and Plant Pathology, Mississippi State University, Starkville, USA; 2 Heart and Vascular Department, The Hattiesburg Clinic, Hattiesburg, USA

**Keywords:** stretch-induced arrhythmias, salt, triggers, atrial tachycardia, atrial fibrillation

## Abstract

A variety of potential triggers of atrial fibrillation (AF) have been reported, including alcohol consumption, caffeine, exercise, and stress. Vagal AF triggers include gastrointestinal factors such as the amount of food consumed, types of foods, and gas and bloating. In this case report, detailed data of seven dietary and behavioral factors (many vagal) believed to be possible atrial tachycardia (AT) or AF triggers in a single patient with paroxysmal AT/AF were recorded. Episodes of AT and AF were recorded in the patient using a Medtronic loop recorder and analyzed by a cardiac electrophysiologist. To evaluate these potential triggers of AT/AF events, a general linear model with binomial family error distribution was used to fit the data. Then, a stepAIC function from the MASS package in R was used to perform a stepwise model selection using AIC (Akaike information criterion). The analysis only identified the amount of salt intake and the use of polyethylene glycol 3350 as predictors of AT/AF, and high salt intake was the only factor significantly associated with the onset of AT/AF (*P <* 0.05). Thus, salt intake may trigger AT/AF in ways other than via hypertension.

## Introduction

In a recent article by Marcus et al., various triggers of paroxysmal atrial fibrillation (AF) were evaluated, including caffeine, reduced sleep, exercise, alcohol, lying on the left side, large meals, and consumption of cold food/drink [1]. Interestingly, in that study, the only trigger significantly heightening the risk of AF events was alcohol. In the vaguely defined condition termed vagal AF, gastrointestinal triggers are often reported, including amount of food consumed, types of foods, and gas and bloating [2-3], but these may be anecdotal or poorly supported by evidence. There is some scientific evidence that episodes of atrial tachycardia (AT) can trigger AF [4-7]. Brugada et al. reported that fast atrial rates can trigger AF, and patients with documented or clinically suspected paroxysmal atrial or atrioventricular reentrant tachycardias can occasionally present with AF [6]. To further explore possible triggers for AT and AF, we analyzed behavioral and dietary factors in a patient with paroxysmal AT/AF over a six-month period.

## Case presentation

The first author of this report (JG) has a 10-yr history of symptomatic paroxysmal AF, almost always preceded by easily detectable 5-10 sec duration AT, serving as precursors to these AF episodes. Confirmation of AT/AF episodes was made by marking them with the wand of a Medtronic Loop Recorder (Dublin, Ireland) and subsequent analysis by the third author (MB). From May 21, 2020, through November 18, 2020 (six months), the author kept detailed daily records of seven dietary and behavioral factors (many vagal) believed to be possible AT/AF triggers (Table 1).

**Table 1:** Dietary and behavioral factors evaluated as possible triggers of atrial tachycardia (AT) and atrial fibrillation (AF) Results of the stepAIC function using the model: glm (h~a+b+c+d+e+f+g, family="binomial," h being the dependent variable – episodes of AT/AF) ^1^1350 mg (1), 700 mg (2), 1050 mg (3), 1400 mg (4),2 1750 mg (5), 2100 mg (6), 2450 mg (7), and >2800 mg (8). ^2^The American Heart Association recommends an ideal limit of 1500 mg sodium per day for most adults. *Significant at the p<.05 level.

Predictor Variables	Dependent Variable: Episodes of AT/AF							
								Full Model			Final Model				
	Estimate	Std Error		Estimate	Std Error	Z Value	Pr (>[Z])	
	Salt ^1,2^	-0.883	-0.377		-0.755	-0.35	-2.14	0.0328*	
Assessed on a scale 1-8 (8 max)								
	Stress			-0.353					-0.481		---	---	---	---	
Assessed on a scale 1-10 (10 max)														
	Statin use	-0.49	-0.823			---	---	---		---					
Daily simvastatin 40 mg, yes or no										
	Gas			0.456	-0.323						---	---	---	---	
Assessed on a scale 1-10 (10 max)														
	Polyethylene glycol 3350	1.21	-0.835				1.158	-0.82	1.417	0.156					
Osmotic laxative, as needed yes or no										
	V8^®^ vegetable juice (8 oz)			-1.292	-1.307						---	---	---	---	
Daily as a source of potassium yes or no														
	Constipation	0.359	-0.335				---	---	---	---					
Assessed on a scale 1-10 (10 max)										
	Intercept			0.703	-2.906						-0.516	-1.606	-0.321	0.7481	
Observations	181			181				
Log likelihood	-32.205			-34.651				
Akaike Inf. Crit.	80.41			75.302				

The patient does not have hypertension, and during the six-month period, no changes were made in the patient’s treatment for AF - 150 mg propafenone bid. To evaluate these potential triggers of AT/AF events, the data were blinded and given to a statistician (second author) for analysis using a general linear model with binomial family error distribution to fit the data. We then used the stepAIC function from the MASS package in R (Venables, W. N. & Ripley, B. D. (2002) Modern Applied Statistics with S. Fourth Edition. Springer, New York) to perform a stepwise model selection using AIC (Akaike information criterion) [8]. The stepAIC function starts with a full (or null) model and uses stepwise regression as a search method for feature selection, minimizing the AIC value and ending with a final set of features.

The stepAIC function analysis only identified the amount of salt intake and the use (or not) of polyethylene glycol 3350 as predictors of AF precursors. On eight out of the 10 days the patient experienced an AT/AF, he had taken polyethylene glycol 3350; and on seven of those days, he had consumed more salt than usual (greater than 4 on a scale of 8, see Table 1 for values). Only high salt intake was significantly associated with the onset of AT/AF (*p* < 0.05).

## Discussion

In this blinded study, only sodium intake was statistically significant (*p*<0.0328) as a trigger for AT/AF. Interestingly, our analysis showed that emotional stress in this patient was not significantly associated with AT/AF, a finding consistent with vagal AF. These data suggest that salt should be considered a trigger for AT/AF, and therefore its intake limited to reduce or mitigate episodes of paroxysmal AF. The negative side effects of salt on cardiac disease are debatable; however, high sodium concentrations may stiffen endothelial cells, perhaps leading to inflammation and vascular remodeling [9]. The mechanical environment of the heart is physiologically dynamic, which inherently affects its activity. We propose that the mechanism for salt as an AT/AF trigger is sodium mediating the mechano-electrical transduction processes underlying the genesis of stretch-induced arrhythmias [10]. Excessive salt could cause stretch-activated channels to fire and trigger AT/AF by altering the length and/or tension of cardiac tissues [10-11]. Levi et al. reported that a rise of intracellular sodium concentration results in an increase of intracellular calcium via the important and influential sodium/calcium exchange mechanism in the cell membrane of cardiac muscle cells [12]. A rise of intracellular calcium modulates the activity of a number of sarcolemmal ion channels and affects the release of intracellular calcium from the sarcoplasmic reticulum, all of which may be involved in causing arrhythmias. Further, Levi et al., in experiments using working rat heart preparations, found that a rise in sodium might also lead to wall-stress-induced arrhythmias [12].

## Conclusions

Excessive sodium from salt and other sources causes fluid retention, and in some people, hypertension, which is a known risk factor for AF. However, salt intake may trigger AT or AF in ways other than via hypertension, such as by increasing intracellular calcium levels through the sodium/calcium exchange mechanism in cardiac tissue, which then affects the release of intracellular calcium from the sarcoplasmic reticulum, leading to arrhythmias. Further, sodium affects the mechano-electrical environment of the heart, possibly causing stretch-induced arrhythmias due to changes in cell length or tension. Controlled studies with multiple patients are needed to ascertain the role of salt as a trigger for paroxysmal AT/AF.

## References

[1] Marcus GM, Modrow MF, Schmid CH (2022). Individualized studies of triggers of paroxysmal atrial fibrillation: the I-STOP-AFib Randomized Clinical Trial. JAMA Cardiol.

[2] Carpenter A, Frontera A, Bond R, Duncan E, Thomas G (2015). Vagal atrial fibrillation: what is it and should we treat it?. Int J Cardiol.

[3] Hansson A, Madsen-Härdig B, Olsson SB (2004). Arrhythmia-provoking factors and symptoms at the onset of paroxysmal atrial fibrillation: a study based on interviews with 100 patients seeking hospital assistance. BMC Cardiovasc Disord.

[4] (2022). Atrial tachycardia. Johns Hopkins Health Information Series. https://www.hopkinsmedicine.org/health/conditions-and-diseases/atrial-tachycardia#:~:text=Atrial%20tachycardia%20(AT)%20is%20a,atria%20to%20beat%20too%20quickly..

[5] Hamer ME, Wilkinson WE, Clair WK, Page RL, McCarthy EA, Pritchett EL (1995). Incidence of symptomatic atrial fibrillation in patients with paroxysmal supraventricular tachycardia. J Am Coll Cardiol.

[6] Brugada J, Mont L, Matas M, Navarro-López F (1997). Atrial fibrillation induced by atrioventricular nodal reentrant tachycardia. Am J Cardiol.

[7] Haissaguerre M, Marcus FI, Fischer B, Clementy J (1994). Radiofrequency ablation in unusual mechanisms of atrial fibrillation: report of three cases. J Cardiovasc Electrophysiol.

[8] Venables WN, Ripley BD (2002). Modern Applied Statistics with S.

[9] Paar M, Pavenstädt H, Kusche-Vihrog K, Drüppel V, Oberleithner H, Kliche K (2014). Endothelial sodium channels trigger endothelial salt sensitivity with aging. Hypertension.

[10] Haïssaguerre M, Marcus FI, Fischer B, Clémenty J (1994). Radiofrequency catheter ablation in unusual mechanisms of atrial fibrillation. Report of three cases. J Cardiovasc Electrophysiol.

[11] Kohl P, Hunter P, Noble D (1999). Stretch-induced changes in heart rate and rhythm: clinical observations, experiments and mathematical models. Prog Biophys Mol Biol.

[12] Levi AJ, Dalton GR, Hancox JC (1997). Role of intracellular sodium overload in the genesis of cardiac arrhythmias. J Cardiovasc Electrophysiol.

